# Agricultural and Agro-Industrial Residues as Sustainable Sources of Next-Generation Biomedical Materials: Advances, Challenges, and Perspectives

**DOI:** 10.3390/life15121908

**Published:** 2025-12-13

**Authors:** Stefania Lamponi, Roberta Barletta, Annalisa Santucci

**Affiliations:** 1 Department of Biotechnology, Chemistry & Pharmacy, University of Siena, Via Aldo Moro 2, 53100 Siena, Italy; 2 SienabioACTIVE, University of Siena, Via Aldo Moro 2, 53100 Siena, Italy; 3 ARTES 4.0, Viale Rinaldo Piaggio 34, 56025 Pontedera, Italy

**Keywords:** agro-industrial residues, sustainable biomaterials, tissue engineering, drug delivery systems, circular bioeconomy, wound healing applications, antimicrobial therapies, Biosensors, implant coatings, green chemistry approaches

## Abstract

Agricultural and agro-industrial residues are increasingly recognized as sustainable, low-cost feedstocks for high-performance biomedical materials. This review critically examines the translational potential of polysaccharides, proteins, inorganic compounds, and phytochemical-rich extracts derived from agro-waste, highlighting their chemical features, structure–function relationships, and application-specific readiness. Polysaccharides such as nanocellulose, pectin, and chitosan emerge as the most advanced biopolymer platforms, particularly in wound healing, drug delivery, and 3D-printed scaffolds. Protein-derived materials—including collagen, gelatin, keratin, and soy protein—show strong promise in regenerative medicine, though challenges in mechanical stability and batch reproducibility remain. Inorganic phases such as hydroxyapatite and silica obtained from eggshells, rice husk ash, and marine shells demonstrate high bioactivity, with dental and bone applications approaching clinical translation. Finally, fruit-residue phytochemicals provide multifunctional antioxidant and antimicrobial enhancements to composite systems. By integrating material chemistry, processing strategies, and translational considerations, this review outlines the current state, challenges, and future perspectives for advancing agro-waste-derived biomaterials within a circular bioeconomy.

## 1. Introduction

The transformation of agricultural and agro-industrial residues (agro-residues/agro-waste) into biomaterials for biomedical use is a rapidly expanding research field that bridges sustainable development and healthcare innovation [1]. While conventional clinical materials such as metals, ceramics, and synthetic polymers remain widely employed, they often present drawbacks including limited biodegradability, potential cytotoxicity, high production costs, and negative environmental impacts [2,3]. These issues have intensified the search for renewable and biocompatible alternatives, and agro-residues [4,5], produced in massive quantities worldwide, are increasingly recognized as valuable feedstocks [6,7,8].

Far from being simple waste streams, agro-residues such as chestnut burrs, *Crocus sativa*, *Cannabis sativa*, cherry waste, crop stalks, fruit peels, shells, and fish by-products are naturally rich in polysaccharides, proteins, lipids, minerals, and bioactive compounds [6,9,10]. These chemical building blocks can be processed into high-value biomaterials with adjustable physicochemical properties, structural integrity, and biological activity [11,12]. Importantly, their conversion into biomedical products not only reduces environmental burdens but also aligns with the principles of a circular bioeconomy, where waste is reimagined as a sustainable resource for advanced medical technologies [13,14,15,16,17,18,19,20].

This chemical diversity of agro-residue content enables the design of materials tailored to specific characteristics and activities. For example, nanocellulose from lignocellulosic biomass can mimic the extracellular matrix, collagen and gelatin from marine or slaughterhouse residues can promote angiogenesis and cell proliferation, and lignin-based composites can improve mechanical performance while maintaining biodegradability [21,22,23]. Likewise, polysaccharides such as cellulose, hemicellulose, alginate, and pectin can be processed into hydrogels, nanoparticles, or films able to adsorb and release drugs [24,25,26], whereas inorganic components like silica from rice husks or hydroxyapatite from eggshells can be integrated to reinforce structural strength and enhance bioactivity [27]. Chitosan shows antimicrobial and hemostatic effects [28], while cellulose- and pectin-based hydrogels maintain a moist environment that fosters tissue regeneration. When combined with mineral-rich residues, these formulations can yield multifunctional dressings with both protective and therapeutic functions [29,30].

Such versatility explains why agro-residue-derived biomaterials are being investigated across multiple biomedical domains. In tissue engineering, they may serve as scaffolds that guide cell adhesion, proliferation, and differentiation [31]. In drug delivery, they act as carriers enabling controlled release of active pharmaceutical ingredients (APIs), thereby improving therapeutic efficacy and patient compliance [32]. In wound management, advanced dressings developed from these materials provide protection, regulate moisture, deliver antimicrobial activity, and actively promote tissue repair [33]. Their potential also extends to diagnostics: nanocellulose, lignin, and conductive biopolymers from agro-residues have been incorporated into biosensors with high sensitivity and selectivity [34,35,36,37].

Implantable devices are another field where agro-residues show great promise. While synthetic hydroxyapatite, metal alloys, and polymers have long been standard choices, they often require energy-intensive processing and non-renewable raw materials and may show limited bioactivity or poor degradation compatibility with host tissues [38,39]. In contrast, composites made from agro-residue-derived hydroxyapatite, collagen, or chitosan exhibit mechanical stability, osteoconductivity, and reduced immune rejection, making them attractive alternatives for bone, dental, and soft-tissue applications [40,41,42,43].

Taken together, agricultural and agro-industrial residues represent a vast and underutilized reservoir of biopolymers that can be converted into biocompatible and biodegradable materials [44,45,46]. Beyond their biomedical functionality, their valorization supports circular bioeconomy strategies, reduces environmental impact, and provides renewable alternatives to traditional synthetic platforms [47,48].

Despite the growing number of studies on waste-derived biomaterials, existing reviews typically focus on individual material classes (e.g., polysaccharides, proteins, or inorganic phases) or on specific applications such as wound dressings or bone scaffolds. A comprehensive and comparative evaluation across all major biomaterial categories derived from agro-residues is still lacking. This review addresses this gap by systematically analyzing the structure–function relationships, processing methods, application domains, and translational maturity of polysaccharides, proteins, inorganic compounds, and phytochemical-rich extracts obtained from agricultural waste. By integrating these perspectives within the broader context of circular bioeconomy, our work provides a unified and critical assessment of the field, offering a roadmap for future research and for the clinical translation of sustainable biomaterials.

## 2. Agro-Waste-Derived Polysaccharides in Biomaterials Development

Polysaccharides are among the most abundant biomolecular constituents of agricultural and agro-industrial residues, and their chemical diversity makes them especially attractive for biomedical material design. These natural polymers combine biocompatibility, biodegradability, and adjustable physicochemical traits, allowing their conversion into advanced formats including hydrogels, nanofibers, aerogels, membranes, and nanoparticles [49,50].

A major advantage of polysaccharide-based biomaterials is their capacity to replicate extracellular matrix (ECM) features, supporting cellular attachment, proliferation, and differentiation, while also functioning as delivery vehicles for therapeutic molecules with controlled release profiles. Their reactive functional groups further enable chemical modification or blending with proteins and inorganic phases to reinforce mechanical strength, enhance bioactivity, and impart responsiveness to biological environments. Consequently, polysaccharides obtained from agro-waste are gaining recognition as sustainable and economical substitutes for petroleum-derived polymers in applications ranging from scaffolds and wound dressings to drug delivery systems and biosensor development [51,52].

### 2.1. Cellulose Nanostructures

Cellulose nanostructures derived from agro-waste, particularly cellulose nanofibrils (CNFs) and cellulose nanocrystals (CNCs), have attracted great attention across several biomedical domains, yet their degree of readiness for clinical translation varies considerably. In tissue engineering, CNF-based scaffolds, especially when combined with hydroxyapatite (HA), stand out as the most advanced approach [53,54]. Their nanoscale architecture closely mimics the extracellular matrix; they support osteogenic differentiation, and their mineralized composites provide both mechanical reinforcement and osteoconductivity [53,54,55,56,57,58]. These features directly address the requirements of bone repair, including load-bearing capacity and integration with host tissue, placing CNF/HA composites closer to clinical application than other cellulose nanostructures.

By contrast, CNCs have been extensively investigated for drug delivery, where functionalization with bioactive ligands or stimuli-responsive groups allows for site-specific and controlled release. Systems such as pH-triggered carriers for 5-fluorouracil in colon cancer models highlight the versatility of CNCs [59,60]. Nevertheless, these drug delivery applications remain mostly confined to in vitro or small-animal studies. Issues such as large-scale production, reproducibility, and long-term safety remain unresolved, delaying their transition toward clinical use compared to CNF/HA composites in bone repair.

Wound healing applications appear more mature, since nanocellulose hydrogels and aerogels already demonstrate several properties that meet clinical needs. They combine moisture retention, exudate absorption, and mechanical stability with the possibility of antimicrobial functionalization through silver, zinc oxide, or phenolic extracts [61]. Rice husk-derived hydrogels and nanocellulose/ZnO composites, for example, have shown antibacterial activity while remaining compatible with fibroblasts. Because wound dressings generally face less stringent regulatory barriers than implants, these products may represent one of the most immediate translational opportunities for nanocellulose [62].

Electrochemical biosensors represent another promising area, as nanocellulose provides a flexible and porous platform for enzyme or receptor immobilization, while integration with conductive additives such as graphene or metallic nanoparticles enhances electron transfer. Composites of CNFs and graphene have been used to anchor acetylcholinesterase for the detection of organophosphate pesticides, achieving sensitivity comparable to conventional supports [63]. Moreover, electrospun cellulose fibers have enabled the fabrication of lightweight, conformable epidermal sensors for monitoring sweat electrolytes, lactate, and glucose [64]. Although these examples highlight strong potential for wearable devices, most prototypes remain in laboratory or preclinical stages, and challenges related to stability, reproducibility, and large-scale manufacturing must be overcome before clinical deployment.

Applications in soft tissue engineering and temporary implants are still at an early stage. Nanocellulose hydrogels, owing to their high-water content, tunable degradation, and biocompatibility, have been investigated for cartilage repair and as depots for local drug delivery. However, their mechanical performance under the dynamic conditions of soft tissues and their long-term resorption profiles require further optimization. As a result, these systems remain less mature compared to wound dressings or bone scaffolds [65].

Taken together, the comparison suggests that CNF/HA composites for bone regeneration [56] and nanocellulose-based wound dressings [62] are the closest to clinical application, thanks to their demonstrated bioactivity, mechanical suitability, and alignment with regulatory requirements. Nanocellulose-based biosensors represent an intermediate stage, with promising prototypes that have yet to undergo validation in real-world scenarios [34,37]. CNC-based drug delivery systems and nanocellulose hydrogels for cartilage and soft-tissue repair remain at an earlier stage of development, with major challenges in reproducibility, scalability, and regulatory approval still to be addressed (Table 1) [66].

### 2.2. Pectin

Pectin extracted from agro-waste has been investigated across several biomedical applications, yet the maturity of these applications differs considerably. In the field of wound healing, pectin hydrogels and films derived from citrus or passion fruit residues have demonstrated bioadhesion, antimicrobial activity, and accelerated wound closure in animal burn models [67,68]. These properties are highly consistent with the clinical requirements for wound dressings, particularly since such devices face fewer regulatory barriers than implantable systems. The fact that pectins co-extract bioactive phytochemicals, which enhance antioxidant and antimicrobial activity, further supports their translational potential. Among the examples reported, orange peel hydrogels and passion fruit pectin–chitosan films appear particularly close to clinical application because their multifunctional performance competes favorably with commercial alternatives [67,68].

In drug delivery, pectin microspheres designed for colon-specific release of 5-fluorouracil take advantage of selective degradation by colonic microbiota to achieve targeted delivery [69]. This strategy effectively increases local drug concentration while minimizing systemic toxicity, which is highly desirable in cancer therapy. Similarly, pectin–chitosan nanoparticles for oral insulin delivery have shown improved intestinal absorption and glycemic control in preclinical models [70], while pectin nanoparticles have been used for oral vaccine delivery to enhance mucosal immune responses [71]. Although these findings are compelling, such approaches remain largely at the preclinical stage, with challenges related to reproducibility, large-scale formulation, and regulatory approval still unresolved. Therefore, while colon-targeted microspheres and oral insulin carriers highlight the versatility of pectin-based systems, they are not yet as close to clinical translation as wound healing applications.

In tissue engineering, particularly for cartilage repair, pectin-based hydrogels have been investigated for their mucoadhesive properties and ability to retain bioactive molecules [72]. However, their intrinsic limitations, such as rapid enzymatic degradation and poor mechanical stability, reduce their standalone suitability. The development of pectin–chitosan polyelectrolyte complexes partially addresses these shortcomings by improving stability, antimicrobial properties, and cell adhesion [73]. Even so, the lack of long-term in vivo data and the complexity of cartilage regeneration delay their translational readiness compared to wound healing products.

Taken together, the evidence suggests that pectin-based wound dressings are the most advanced toward clinical application, thanks to demonstrated antimicrobial activity, strong bioadhesion, and effective performance in animal models [74]. Drug delivery systems, including oral insulin carriers, oral vaccines, and colon-specific chemotherapy microspheres [69,70], represent highly innovative uses but remain at an earlier stage of development, with regulatory and manufacturing challenges yet to be resolved. Applications in cartilage tissue engineering are promising but limited by intrinsic material weaknesses, despite improvements obtained through polyelectrolyte complexation with chitosan [29]. Thus, while pectin demonstrates remarkable versatility, its clinical translation will depend on how effectively these limitations are addressed through composite design and scalable production strategies (Table 1).

### 2.3. Chitosan

Chitosan obtained from agro-waste represents one of the most versatile biopolymers under investigation for biomedical use, but the degree of translational maturity differs among its applications. In drug delivery, the mucoadhesive and permeation-enhancing properties of chitosan have been exploited in several routes of administration [75,76]. Nanoparticles derived from mushroom residues for oral insulin delivery, for example, demonstrate improved stability in acidic environments and enhanced intestinal uptake [77]. While these preclinical studies are promising, clinical translation remains challenging because of the complexity of oral peptide delivery and the variability of gastrointestinal absorption. Similarly, chitosan-based ocular carriers for antibiotics have demonstrated the ability to extend corneal residence time and reduce dosing frequency [78,79], an advantage that aligns well with the clinical needs in ophthalmology. Given the relatively low regulatory barrier for ophthalmic formulations compared to systemic therapies, ocular delivery systems may be closer to real clinical application than oral insulin platforms.

The use of chitosan in nucleic acid delivery is conceptually attractive, as its electrostatic interaction with DNA and siRNA offers a biodegradable alternative to viral vectors [80,81]. However, despite good in vitro performance, the clinical pathway for gene delivery remains complex, requiring reproducible large-scale formulations, controlled release profiles, and extensive safety validation. As such, nucleic acid delivery applications remain in the early preclinical stage.

In wound healing, chitosan appears more advanced, since it promotes clot formation, angiogenesis, and granulation, all of which are highly relevant to tissue repair. Composite dressings incorporating rice husk-derived cellulose and zinc oxide have demonstrated enhanced antimicrobial activity and effective exudate management [82,83,84]. These multifunctional properties, combined with the regulatory feasibility of wound dressings compared to implantable materials, suggest that chitosan-based wound products are among the most clinically mature applications. The incorporation of bioactive phytochemicals, such as polyphenols from grape pomace, further adds antioxidant and antibacterial functions, expanding their therapeutic potential [85].

Chitosan-based hydrogels derived from agricultural residues are also being studied as soft tissue fillers, wound-healing matrices, and short-term implants. Their high-water content, tunable degradation, and biocompatibility make them attractive for cartilage regeneration or as local drug delivery depots. Nevertheless, limitations in mechanical stability under physiological stresses and the absence of long-term in vivo data still hinder their clinical translation. Compared with wound dressings and ocular carriers, these soft-tissue and implantable systems remain less mature [86].

Overall, the data suggest that wound-healing applications and ocular drug delivery carriers are the closest to clinical adoption, supported by strong biological activity and relatively favorable regulatory pathways [78,79,82,83,84]. Oral peptide and gene delivery systems, though innovative, remain at a preclinical stage due to challenges in reproducibility, safety, and large-scale production [77,80,81]. Short-term implants and soft-tissue fillers based on chitosan hydrogels occupy an intermediate position, with promising functionality but unresolved issues regarding mechanical robustness and long-term biocompatibility (Table 1).

### 2.4. Hybrid Systems

The integration of polysaccharides with other natural polymers and inorganic materials has produced a wide class of hybrid and nanostructured systems that aim to combine the biological functionality and tunable chemistry of biopolymers with the mechanical strength, bioactivity, or controlled-release properties contributed by complementary components. A representative example is offered by pectin–chitosan composites, which have been explored as extrusion-based bioinks for articular cartilage repair. These blends improve printability, enable reproducible extrusion, and generate constructs with cytocompatibility and controlled degradation, supporting the survival of encapsulated cells and the induction of early chondrogenic markers in vitro [87]. Similarly, cellulose-based matrices reinforced with silica nanoparticles, including silica obtained from rice husks, have demonstrated enhanced stiffness, improved resistance to deformation, and higher encapsulation efficiency of drugs. The sustainable sourcing of silica from agricultural waste also offers cost and environmental advantages, although batch-to-batch reproducibility remains a concern [88].

Additional case studies include nanocellulose- or cellulose-derivative bioinks combined with alginate, gelatin or hyaluronic acid, which exhibit shear-thinning behavior, high shape fidelity after printing, and mechanical properties suitable for musculoskeletal applications. In vivo experiments in small animals have shown improved cartilage and bone regeneration compared with polymer-only controls, positioning these systems as promising candidates for clinical translation [89,90]. Inorganic–polysaccharide hybrids have also been designed for theranostic applications: for example, chitosan, dextran, or alginate matrices loaded with gold, silica, or iron oxide nanoparticles enable simultaneous drug delivery and imaging or therapy. These systems exploit the stimuli-responsiveness of the inorganic component while relying on the polysaccharide matrix to provide biocompatibility and biodegradability [91].

When comparing these approaches, nanocellulose–alginate and pectin–chitosan bioinks appear closest to clinical application because they build upon biopolymers with established safety records and regulatory precedents, and because they show manufacturability compatible with current biofabrication processes. In contrast, cellulose–silica composites may find faster translation in dental and orthopaedic applications, where regulatory pathways are more defined and mechanical reinforcement is a primary need [24,68,73,87]. Nonetheless, hybrids that incorporate significant amounts of inorganic nanoparticles still face important barriers, including incomplete understanding of long-term biocompatibility, difficulties in ensuring reproducibility of bio-derived fillers, and challenges associated with sterilization processes. Systems that rely on cell-laden constructs also face an additional layer of regulatory complexity due to the need for GMP-compliant handling and quality control [26].

Overall, hybrid systems represent a powerful strategy to achieve a balance between printability, bioactivity, and mechanical stability. Their translational trajectory, however, depends strongly on material selection, standardization of processing methods, and long-term in vivo validation. The most promising near-term prospects are hybrids that incrementally modify well-characterized polysaccharides, while more complex inorganic–polysaccharide systems remain at an earlier stage of development (Table 1) [26].

### 2.5. Critical Comparative Analysis

When comparing waste-derived polysaccharides, distinct trade-offs emerge regarding microstructure and mechanical performance. Nanocellulose (CNF/CNC) exhibits a highly crystalline, fibrous microstructure that offers superior tensile strength and stiffness, making it the preferred reinforcement agent for load-bearing composites. However, it lacks intrinsic cellular interaction motifs; cells attach primarily through physical entrapment or non-specific electrostatic forces unless chemically modified [58]. In contrast, chitosan possesses a semi-crystalline structure with cationic amine groups that actively promote biocompatibility and hemostasis by interacting with negatively charged cell membranes and blood components [82]. Pectin, while mechanically weaker due to its amorphous, gel-like structure, offers unique microstructural advantages for soft tissue engineering, forming hydrated networks that closely mimic the viscoelasticity of the native extracellular matrix, though it requires crosslinking to match the degradation rates of cellulose-based systems [24,28].

## 3. Agro-Waste-Derived Proteins in Biomaterials Development

Proteins derived from agro-industrial by-products represent a high-value class of biomaterials that offer distinct advantages over polysaccharide-based systems, particularly regarding biological signaling and enzymatic biodegradability [92]. Unlike polysaccharides, which primarily provide structural scaffolding, proteinaceous materials such as collagen, gelatin, keratin, and soy protein naturally contain bioactive amino acid sequences (e.g., RGD motifs) that actively promote cell adhesion, migration, and differentiation [92]. While historically less utilized than cellulose due to more complex extraction requirements, waste-derived proteins are rapidly gaining traction as a sustainable alternative to mammalian proteins, addressing ethical and supply chain concerns associated with bovine or porcine sources [92]. These biopolymers offer a versatile platform for scaffold and carrier development; their amphiphilic nature and abundance of reactive side groups allow for diverse functionalization strategies, including crosslinking and bioconjugation. By recovering proteins from sources such as slaughterhouse residues, fish scales, and plant-oil cakes, researchers can engineer materials that mimic the native extracellular matrix (ECM) more closely than synthetic or polysaccharide-only counterparts, positioning them at the forefront of regenerative medicine [92].

### 3.1. Collagen and Gelatin

Collagen, the most abundant structural protein in the extracellular matrix, is a widely investigated biopolymer for biomedical applications. It can be extracted from animal-derived waste such as slaughterhouse residues and fish scales using acid extraction or enzymatic digestion, which preserves the native triple-helix structure and bioactive motifs essential for biological function [93,94]. These motifs, particularly RGD sequences, promote cell adhesion, migration, and angiogenesis, making collagen a favorable material for scaffolds in regenerative medicine [95]. Collagen from waste streams has been processed into porous sponges, hydrogels, and electrospun nanofibers, often employed as carriers for antibiotics or growth factors to enhance tissue repair [96,97].

Several studies have highlighted the use of collagen-based biomaterials in wound healing and tissue engineering. For instance, electrospun collagen nanofibers loaded with antimicrobial peptides or silver nanoparticles demonstrated strong antibacterial activity while supporting fibroblast proliferation, showing promise as advanced wound dressings [98]. Similarly, collagen–hydroxyapatite composites have been extensively investigated as bone graft substitutes, with multiple preclinical studies confirming their osteoconductivity and integration into host tissue [99]. A further innovative source of collagen is the eggshell membrane (ESM), naturally rich in collagen and glycosaminoglycans, which also possesses intrinsic anti-inflammatory and antimicrobial properties [100]. Composite dressings combining ESM with calcium carbonate or bioglass leverage both the membrane’s mechanical integrity and the bioactivity of inorganic ion release, accelerating wound closure in animal models and improving bacterial clearance [101].

Gelatin, produced by partial hydrolysis of collagen, offers enhanced solubility and ease of processing compared to native collagen and is widely used in biomedical research and applications [102]. However, gelatin scaffolds and carriers are mechanically weaker and undergo rapid enzymatic degradation, necessitating crosslinking strategies (e.g., genipin, carbodiimide, enzymatic crosslinkers) to extend stability in vivo [103]. Despite this limitation, gelatin’s versatility has supported its use in drug delivery and regenerative medicine. Gelatin nanoparticles derived from fish scale collagen, for example, have been loaded with doxorubicin, and surface modification with folic acid enabled tumor-targeted delivery, significantly increasing antitumor efficacy in preclinical cancer models [104]. In musculoskeletal regeneration, gelatin microspheres encapsulating bone morphogenetic proteins (BMPs) have enabled sustained release, accelerating bone repair and demonstrating superior outcomes compared with bolus delivery in vitro [105]. Moreover, gelatin nanofibers derived from fish scales and loaded with ciprofloxacin have achieved accelerated wound closure in infected rat models, illustrating the potential of gelatin-based antibiotic carriers in wound care [106,107].

Other case studies underscore the flexibility of gelatin for tissue engineering. Injectable gelatin methacryloyl (GelMA) hydrogels, which can be photopolymerized in situ, have emerged as leading candidates for cartilage and cardiac tissue engineering because of their tunable stiffness, printability, and ability to support angiogenesis [108]. GelMA-based bioinks are already being tested in large-animal models, and some have advanced toward early-phase clinical trials [109]. Similarly, gelatin–chitosan blends have been investigated for nerve regeneration, where their balanced degradation rate and support of Schwann cell activity contribute to peripheral nerve repair [110].

**Figure 1:**
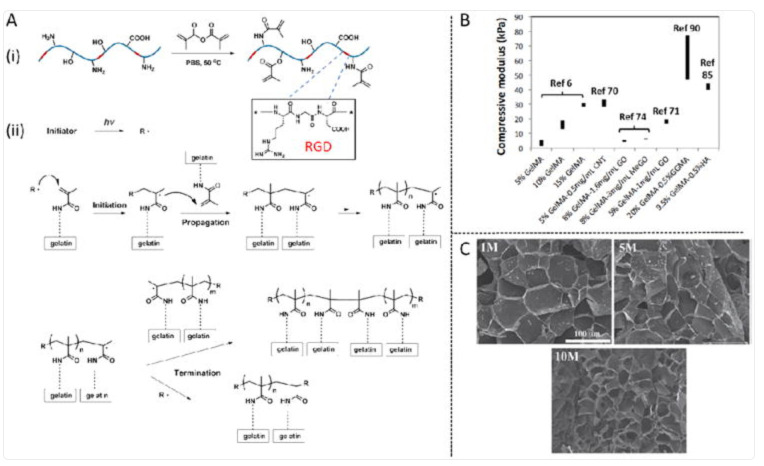
Schematic Synthesis and characterization of GelMA hydrogels. (**A**) Scheme for preparation of photocrosslinked GelMA hydrogel. (i) Reaction of gelatin and methacrylic anhydride for grafting of methacryloyl substitution groups. The modification occurs at primary amine and hydroxyl groups. The RGD domains are illustrated as red segments along the GelMA chains, and their chemical structure is depicted within the inset. (ii) Representative reactions during the photocrosslinking of GelMA to form hydrogel networks. Free radicals are generated from photoinitiators, which initiate the chain polymerization of the methacryloyl substitutions. Propagation occurs between methacryloyl groups located on the same chain and on different chains. Termination occurs between two propagating chains or between one propagating chain and a second radical. Chain transfers and many other minor reactions are not shown, for clarity. (**B**) The compressive modulus reported by several studies on GelMA hydrogels (Nichol et al., 2010; Shin et al., 2012; Shin et al., 2013; Cha et al., 2014; Levet et al., 2014; Shin et al., 2012). (**C**) SEM images of GelMA hydrogels, showing the effect of the degree of methacryloyl substitution on the pore sizes of GelMA hydrogels. Adapted with permission from Yue et al. [108].

When comparing collagen- and gelatin-based systems, collagen scaffolds more closely mimic the native extracellular matrix, offering superior biological signalling and integration potential. However, they often require reinforcement with inorganic fillers or synthetic polymers to achieve sufficient mechanical stability, particularly in load-bearing applications. Gelatin, by contrast, offers easier processing and chemical tunability, making it more adaptable for drug delivery systems, nanoparticles, and microspheres, but requires crosslinking or blending to achieve longevity in vivo. Systems such as GelMA hydrogels (Figure 1) represent a convergence of biological activity and engineering control, providing a platform that is currently among the most advanced toward clinical translation [108].

In terms of translational readiness, collagen–hydroxyapatite composites are already used in clinical practice for bone regeneration and dental applications [99], while collagen dressings enriched with silver or antibiotics are commercially available for wound management [98]. Gelatin-based drug delivery systems, particularly nanoparticles, remain largely at the preclinical stage, with encouraging results in oncology but limited clinical testing to date [104]. GelMA-based scaffolds and bioinks represent the most clinically advanced gelatin derivatives, with ongoing investigations in musculoskeletal and cardiac repair [108,109]. The main limitations of both collagen and gelatin remain batch-to-batch variability (due to source and extraction method), immunogenic risk when derived from animal waste, and challenges in scaling up manufacturing under GMP standards.

Overall, collagen and gelatin provide highly versatile biomaterials with distinct strengths: collagen excels in biological mimicry and integration, whereas gelatin offers superior processability and versatility for drug delivery. While several collagen-based products have already reached the clinic, gelatin-based nanocarriers and advanced derivatives such as GelMA are rapidly progressing and may represent the next wave of clinically relevant biomaterials (Table 2).

To assess the market viability of waste-derived collagen, it is necessary to compare it with established clinical products such as Bio-Gide^®^ (Geistlich Pharma), the current gold standard for resorbable bilayer membranes in Guided Bone Regeneration (GBR). While bovine-derived membranes like Bio-Gide^®^ exhibit a denaturation temperature (Td) of approximately 39 °C, ensuring stability at body temperature, untreated collagen from cold-water fish waste often has a lower Td (approx. 25–30 °C), which can compromise in vivo stability. However, recent advancements in crosslinking fish collagen have successfully raised its thermal stability to levels comparable to mammalian commercial products. Furthermore, unlike bovine sources which carry risks of prion transmission (BSE) and cultural constraints (e.g., Halal/Kosher compliance), marine collagen derived from fish processing by-products offers a “universally acceptable” alternative that meets the safety profile of synthetic hydrogels while retaining the RGD bioactive motifs that synthetics lack [93].

### 3.2. Keratin

Keratin, a structural protein abundantly available from renewable sources such as feathers, wool, hair, and even human hair salon waste, has emerged as a versatile biomaterial due to its unique amino acid composition and abundance of cysteine disulfide bonds. These confer intrinsic bioactivity, redox-responsiveness, and slow degradation properties, making keratin an attractive candidate for biomedical applications [111]. Extraction techniques, including reduction, oxidation, and ionic liquid processing, allow recovery of keratin while retaining bioactive peptide sequences that can promote cell adhesion, proliferation, and differentiation [111,112].

Keratin scaffolds have been fabricated into sponges, hydrogels, and electrospun nanofibers, often enriched with polyphenols or metallic nanoparticles to improve functional properties. For example, keratin/polyphenol composites demonstrate enhanced antioxidant activity and protection against oxidative stress, while keratin–silver nanoparticle systems combine antimicrobial activity with the structural support required for skin regeneration [113,114]. In preclinical wound healing models, these hybrid keratin scaffolds supported fibroblast proliferation, angiogenesis, and re-epithelialization, resulting in faster wound closure compared to untreated controls [112,114]. Additionally, keratin dressings have shown superior moisture retention, which is critical in maintaining a favorable wound microenvironment [111].

Beyond wound care, keratin-based systems have been developed for drug delivery. Keratin nanoparticles, due to their amphiphilic nature and presence of functional groups, can encapsulate both hydrophobic and hydrophilic drugs. Several studies have demonstrated their effectiveness in delivering hydrophobic anticancer agents such as paclitaxel, improving drug solubility, protecting against premature degradation, and achieving controlled release at the tumor site [115,116]. For instance, keratin nanoparticles loaded with paclitaxel not only enhanced drug stability but also prolonged circulation time and increased antitumor efficacy in murine models, suggesting translational potential for oncology applications [115]. In another study, keratin nanoparticles modified with folic acid were able to selectively target cancer cells, thereby reducing off-target toxicity and improving therapeutic outcomes [116].

Recent advances in processing techniques have further broadened keratin’s biomedical potential. Electrospinning of keratin-based nanofibers has resulted in highly porous scaffolds with large surface area and mechanical tunability, making them suitable for skin and nerve regeneration [113]. Moreover, 3D printing approaches allow the creation of patient-specific keratin scaffolds with controlled porosity and degradation rates, a significant step toward personalized regenerative therapies [111,113]. In nerve regeneration, keratin conduits have shown promising results by supporting Schwann cell migration and axonal growth, outperforming some synthetic polymer conduits in preclinical models [117].

When comparing keratin-based systems with collagen and gelatin counterparts, keratin stands out for its natural abundance in waste streams and its inherent antioxidant and antimicrobial properties, which are less pronounced in collagen-based biomaterials. Its slower degradation rate, due to extensive disulfide crosslinking, makes keratin particularly advantageous for applications requiring longer-term scaffolding, such as nerve repair. However, keratin often suffers from poor mechanical strength compared to collagen, necessitating blending with other polymers (e.g., chitosan, polycaprolactone) to achieve clinically relevant performance [111,113].

In terms of translational readiness, keratin dressings and hydrogels for wound healing are closest to clinical application, as they address an immediate clinical need and exploit keratin’s intrinsic biological properties [112,114]. Keratin nanoparticles for cancer drug delivery, while highly promising, remain at the preclinical stage and face hurdles related to large-scale reproducibility, sterilization, and regulatory approval for systemic administration [115]. Electrospun keratin scaffolds and 3D-printed constructs are still experimental, requiring further mechanical optimization and validation in large-animal models before progressing toward clinical translation [111].

Overall, keratin-based biomaterials offer unique advantages in wound healing, antioxidant therapy, and drug delivery, with wound dressings representing the most clinically advanced application. However, limitations including mechanical weakness, batch variability from natural sources, and a lack of standardized extraction protocols remain significant obstacles. Addressing these challenges through hybridization with stronger polymers, scalable processing methods, and rigorous in vivo validation will be essential to unlock keratin’s full translational potential (Table 2).

### 3.3. Soy Protein

Soy protein, obtained from defatted soymeal after oil extraction, represents a renewable, low-cost, and abundant protein source that offers a sustainable alternative to animal-derived proteins. Its molecular structure, rich in globulins (glycinin and β-conglycinin), allows it to be processed into films, foams, fibers, and hydrogels that are biodegradable and biocompatible in physiological environments [118]. Importantly, soy protein contains bioactive peptide sequences that can modulate cell adhesion, proliferation, and differentiation, making it a promising platform for biomedical applications. Functionalization with growth factors, peptide motifs, or inorganic ions further broadens its utility, enabling the development of scaffolds with tailored bioactivity and degradation kinetics [119,120].

Soy protein has been successfully fabricated into porous scaffolds, sponges, and nanofibers suitable for soft tissue engineering. For example, soy protein nanofiber scaffolds created by electrospinning exhibit high surface area-to-volume ratios, favorable porosity, and tunable mechanical strength. These scaffolds have supported fibroblast adhesion and proliferation while displaying degradation rates aligned with tissue regeneration timelines [118,119]. In vivo studies demonstrated that soy protein sponges implanted in rat skin defects accelerate granulation tissue formation and angiogenesis compared with collagen-only controls, highlighting their regenerative potential [121]. Blending soy protein with biopolymers such as chitosan or alginate has also improved mechanical integrity and water resistance, expanding their use in cartilage and skin repair applications [71].

In drug delivery, soy protein nanoparticles and microparticles have been exploited for the encapsulation of hydrophobic and labile drugs. A notable example is soy protein nanoparticles loaded with curcumin, a poorly water-soluble compound with anti-inflammatory and antioxidant properties. Encapsulation significantly improved curcumin’s solubility, stability, and oral bioavailability, while maintaining its antioxidant activity, thereby providing dual benefits in tissue protection and regeneration [122,123]. Similarly, soy protein nanoparticles have been used to deliver doxorubicin, achieving sustained release and reduced systemic toxicity in cancer models [124]. The ability of soy protein carriers to self-assemble and form stable colloidal systems makes them an attractive option for oral and parenteral drug delivery.

Recent research has also expanded soy protein’s role in wound healing. Soy protein–based hydrogels enriched with bioactive peptides or blended with polysaccharides demonstrated enhanced hemostatic properties, faster epithelialization, and reduced scarring in preclinical wound healing models [125]. Moreover, chemical modifications, such as phosphorylation or crosslinking, have been applied to tailor degradation rates and mechanical performance, aligning scaffold stability with tissue-specific healing requirements [118,120].

When compared with collagen, gelatin, and keratin, soy protein stands out primarily for its cost-effectiveness, plant origin, and reduced risk of zoonotic disease transmission or immunogenicity. Its renewable sourcing from agro-industrial residues also makes it highly attractive from a sustainability perspective. However, soy protein scaffolds generally exhibit weaker mechanical properties and higher water sensitivity compared to collagen or keratin, requiring blending or crosslinking for stability in vivo. Furthermore, variability in protein composition depending on soybean cultivar and processing conditions introduces batch-to-batch inconsistencies that could complicate regulatory approval [126].

In terms of clinical translation, soy protein dressings and scaffolds for wound healing are closest to application, particularly when combined with polysaccharides or inorganic fillers to improve their stability and functional performance [120,125]. Drug delivery applications, while promising, remain largely preclinical, with challenges including scale-up of nanoparticle synthesis, sterilization, and controlled release kinetics under physiological conditions. Compared to collagen-based scaffolds, which already have FDA-approved products, soy protein systems are still in earlier stages of development, but they offer unique opportunities as plant-derived, low-cost biomaterials for broad applications in tissue repair and drug delivery [126].

Overall, soy protein-based biomaterials represent a sustainable and versatile alternative to animal-derived scaffolds. Their future success depends on overcoming current mechanical and reproducibility limitations and on generating robust in vivo data to support their regulatory pathway. With these advancements, soy protein could complement or even replace animal-derived proteins in certain biomedical contexts, particularly in wound healing and low-cost regenerative medicine (Table 2).

### 3.4. Hybrid Systems

To overcome the inherent limitations of individual protein-based materials, hybrid scaffolds combining proteins with polysaccharides or inorganic fillers have been extensively investigated. The rationale behind these designs lies in creating composites that unite the biocompatibility and bioactivity of natural proteins with the mechanical strength, printability, or osteoconductivity of reinforcing phases. This synergistic approach can yield materials with improved stability, tailored degradation rates, and multifunctional therapeutic effects, thereby broadening their applicability in tissue engineering and regenerative medicine [127,128].

**Figure 2:**
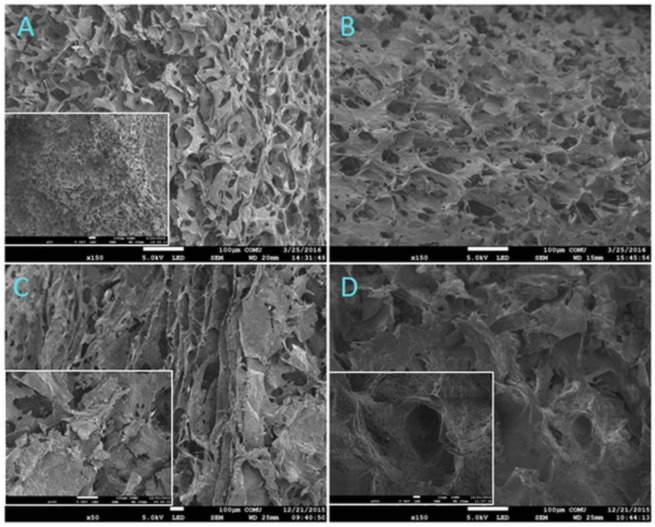
SEM images of collagen (×150, inset ×50) (**A**), collagen-keratin (×150) (**B**), collagen-nHA (×50, inset ×150) (**C**), and collagen-keratin-nHA (×150, inset ×500) (**D**) scaffolds. Scale bars show 100 μm.Adapted with permission from Arslan et al. [128].

A representative example is keratin–hydroxyapatite composites, which leverage keratin’s natural bioactivity and hydroxyapatite’s osteoconductive properties to produce scaffolds suitable for bone tissue regeneration. In vitro studies demonstrated that such composites support osteoblast adhesion, proliferation, and differentiation, while in vivo experiments in rat calvarial defects showed accelerated bone deposition and mineralization compared with keratin-only scaffolds [128]. These findings highlight their potential as alternatives to collagen–hydroxyapatite composites, which already have a longer track record in clinical settings. However, keratin–hydroxyapatite systems still face challenges related to mechanical brittleness and scale-up reproducibility [128] (Figure 2). 

Soy protein has also been successfully reinforced with cellulose nanofibers, resulting in hybrid scaffolds with improved structural integrity, reduced water solubility, and enhanced cytocompatibility. For example, soy protein isolate–cellulose nanofiber composites displayed significantly higher tensile strength and modulus compared with soy protein alone, while maintaining porosity conducive to fibroblast infiltration and proliferation. In wound healing models, these scaffolds promoted granulation tissue formation and vascularization, underscoring their potential in soft tissue repair. Nevertheless, variability in the quality of cellulose derived from agro-industrial sources and the inherent immunogenicity concerns associated with soy proteins remain barriers to clinical translation [127].

Other examples include bacterial cellulose composites reinforced with proteins such as gelatin, silk fibroin, or soy protein. These constructs combine the mechanical robustness of bacterial cellulose with the bioactivity of proteins, resulting in composites with properties closer to those of native soft tissues. Their interconnected porosity promotes cell infiltration, angiogenesis, and tissue integration. In preclinical wound healing models, bacterial cellulose–gelatin dressings reduced inflammation, supported keratinocyte migration, and accelerated epithelialization compared with cellulose-only controls [129]. Furthermore, silk fibroin–bacterial cellulose composites have been explored for vascular grafts and cartilage repair, where their mechanical compliance and biocompatibility were found to outperform synthetic polymeric scaffolds [61].

When critically comparing these systems, keratin–hydroxyapatite composites appear most relevant for bone repair due to their inherent osteoconductivity, yet they lag collagen–hydroxyapatite scaffolds, which are already in clinical use (e.g., bone fillers and dental implants). Soy protein–cellulose hybrids are highly attractive for soft tissue engineering and wound healing, particularly because they are low-cost and plant-derived, reducing ethical and zoonotic risks. However, their mechanical weaknesses and variable degradation profiles require further optimization before regulatory approval [130]. Bacterial cellulose–protein composites are closer to clinical readiness in wound care, as bacterial cellulose has already been used in commercial dressings and the addition of proteins such as gelatin or silk primarily enhances bioactivity without introducing entirely new regulatory hurdles [129,131].

In terms of translational outlook, hybrid systems based on bacterial cellulose and clinically validated proteins (e.g., gelatin, silk) are closest to clinical application, especially in wound healing and soft tissue repair. Conversely, keratin- and soy protein-based hybrids remain promising but face greater challenges related to reproducibility, immunogenicity, and large-scale standardisation. The critical step for all hybrid systems will be to ensure consistent manufacturing, long-term in vivo safety, and compliance with sterilization and regulatory frameworks (Table 2).

### 3.5. Critical Comparative Analysis

The choice between waste-derived proteins depends heavily on the balance between bioactivity and mechanical stability. Collagen and gelatin are superior regarding cellular interactions; they naturally contain RGD (arginine-glycine-aspartic acid) sequences that directly bind to integrin receptors on cell surfaces, promoting rapid adhesion and migration [92]. However, their mechanical properties in isolation are often insufficient for structural applications, and they undergo rapid enzymatic degradation. Conversely, keratin extracted from feathers or wool possesses a robust, crosslinked microstructure due to high cysteine (disulfide bond) content. This results in superior mechanical toughness and slower degradation compared to collagen, yet keratin often displays lower cell infiltration rates due to its denser packing [93]. Soy protein offers an intermediate profile; while its biocompatibility is high, its globular structure requires significant processing (e.g., electrospinning) to generate the fibrous microstructure necessary to guide tissue alignment [112].

## 4. Agro-Waste-Derived Inorganic Compounds in Biomaterials Development

Agricultural and agro-industrial residues serve as an exceptional, yet frequently undervalued, reservoir of inorganic compounds for biomedical engineering. While conventional bioceramics are typically synthesized from geological minerals or pure chemical reagents, waste-derived inorganic phases—such as hydroxyapatite from fish bones or eggshells, and silica from rice husks—possess unique “biogenic” features that synthetic materials often lack [43]. These materials frequently retain a “biological memory” in the form of trace elements (e.g., magnesium, strontium, zinc) naturally incorporated into their crystal lattice during biological formation [88,101,128]. These ionic substitutions have been shown to significantly enhance osteoblast activity and accelerate bone regeneration compared to stoichiometric, synthetic counterparts ]. Furthermore, the extraction of these minerals supports circular bioeconomy principles by replacing energy-intensive mining and synthesis processes with the valorization of abundant waste streams. Consequently, inorganic phases obtained from agricultural residues are increasingly recognized not merely as low-cost fillers, but as advanced, bioactive components critical for the design of next-generation osteoconductive scaffolds and dental materials [132].

### 4.1. Hydroxyapatite from Eggshells

Eggshells and fish bones are abundant sources of calcium carbonate and phosphate that can be converted into hydroxyapatite (HA) by calcination, hydrothermal synthesis, chemical precipitation or combined wet–thermal routes. HA obtained from these biowastes closely resembles the mineral phase of natural bone in both composition and crystallinity when processed appropriately, which explains its widely reported osteoconductive behaviour and suitability for bone-related drug delivery and tissue-engineering applications [128].

Representative examples highlight the translational potential of eggshell-derived HA (ES-HA). In small-animal bone defect models, porous scaffolds and cements prepared from calcined eggshells supported new bone deposition and vascular ingrowth, with histological outcomes comparable to those of commercially available synthetic HA [132]. Injectable apatitic bone cements formulated from eggshell powder have also been shown to set within clinically acceptable times, display good injectability, and deliver antibiotics such as gentamicin or vancomycin with sustained release profiles, a combination that is particularly attractive for the management of osteomyelitis where infection control and bone regeneration must occur simultaneously [101,133]. In implantology, nano-HA synthesized from eggshells has been deposited onto titanium and Ti-alloy substrates to improve osseointegration. Preclinical studies indicate that ES-HA coatings adhere adequately, support osteoblastic attachment and mineralized matrix formation, and can reduce implant micromotion, thus representing promising candidates for dental and orthopaedic applications [134].

Dental uses of ES-HA are especially advanced. Several in vitro and in situ studies, along with pilot clinical investigations, demonstrate that nano-HA derived from eggshells can remineralize bleached or demineralized enamel, occlude dentinal tubules, and be formulated into toothpastes or prophylactic gels. These approaches significantly increase enamel microhardness and surface mineral density, positioning eggshell-derived HA as a low-cost and circular alternative to synthetic nHA for preventive and reparative dental care [135]. Fish-bone-derived HA shows parallel advantages and has been developed for both bone grafts and as a drug carrier. It has been employed as a matrix for bisphosphonate delivery in osteoporosis therapy, improving drug targeting to bone tissue and reducing systemic exposure. Furthermore, doping fish-derived HA with biologically relevant ions such as magnesium or strontium has been explored to tune resorption and stimulate osteogenesis, thereby extending its potential beyond simple osteoconduction [136].

Beyond structural applications, ES-HA has been developed as a multifunctional drug carrier [133]. Nano-HA particles and injectable cements have been loaded with antibiotics to provide local release at infected bone sites, achieving bactericidal concentrations while simultaneously allowing new bone in-growth [137]. Other strategies include doping ES-HA with strontium or magnesium to accelerate osteogenesis in vivo, highlighting the versatility of this material as both a scaffold and a therapeutic reservoir [136].

When compared with fully synthetic HA, ES-HA and fish-derived HA offer distinct advantages, including lower raw-material cost, valorisation of agro-food waste, and chemical compositions that often include trace elements naturally found in bone, which may beneficially influence bioactivity. In dental remineralization and non-load-bearing bone grafts, ES-HA already demonstrates performance comparable to synthetic HA in vitro and in preclinical studies [138]. However, important trade-offs remain. Natural-source HA often shows batch-to-batch variability due to differences in feedstock species, diet, or processing conditions, leading to heterogeneity in crystallinity, resorption rate and mechanical strength. Residual organic matter is another concern, as incomplete calcination or purification can provoke inflammatory responses. Moreover, the surface chemistry and porosity of ES-HA must be carefully controlled: excessive density reduces cellular infiltration, while excessive porosity or rapid resorption compromises mechanical stability.

From a translational standpoint, dental applications such as toothpaste additives, remineralizing gels and prophylactic coatings are the closest to clinical implementation because they require relatively simple regulatory approval compared with implantable drug–device combinations [135]. The next most advanced are acellular bone fillers and coatings, which already show promising preclinical osseointegration and are compatible with existing implant device classifications. More complex constructs, such as antibiotic-loaded injectable cements or load-bearing grafts, remain at an earlier stage of development. These approaches face stricter regulatory requirements, as they must demonstrate reproducible synthesis, predictable degradation and drug release profiles, sterilizability, and long-term safety.

Overall, eggshell-and fish-derived hydroxyapatite represent compelling circular biomaterials that combine osteoconductivity, drug-delivery capability, and dental remineralization potential. While dental and acellular bone applications are closest to clinical use, multifunctional drug-delivery implants and load-bearing scaffolds remain limited by variability, insufficient large-animal studies, and the lack of standardized manufacturing and sterilization protocols. Addressing these challenges through harmonized feedstock characterization, validated synthesis methods, and rigorous preclinical evaluation will be essential to unlock the full clinical potential of these waste-derived HA materials (Table 3).

In the clinical market, synthetic hydroxyapatite (e.g., Engipore^®^ or generic synthetic HA fillers) is valued for its stoichiometry and purity. However, these synthetic products often lack the trace ionic substitutions found in natural bone. Here, eggshell-derived hydroxyapatite (ES-HA) presents a distinct advantage over purely synthetic competitors. Studies comparing ES-HA to commercial synthetic HA controls in bone defect models have shown that ES-HA promotes comparable, and in some cases superior, vascular ingrowth and new bone deposition [132,137]. This enhanced performance is attributed to the “biological memory” of the eggshell source, which naturally retains trace elements such as Magnesium (Mg) and Strontium (Sr) in the crystal lattice—ions known to stimulate osteoblast activity—which must be artificially added to synthetic commercial products [132].

### 4.2. Silica from Rice Husks

**Figure 3:**
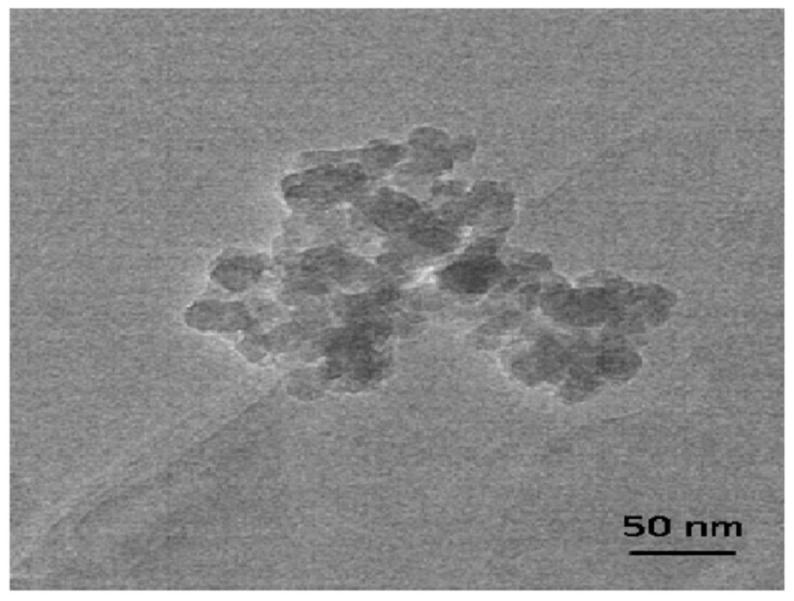
Transmission Electron Microscopy (TEM) images of high-purity nanosilica extracted from rice husk ash, showing uniform particle size distribution. Adapted with permission from Yuvakkumar et al. [88].

Rice husks, an abundant by-product of rice milling, contain approximately 15–20% amorphous silica embedded in a lignocellulosic matrix [62,88]. Through processes such as controlled combustion, alkaline extraction, sol–gel synthesis and chemical etching, rice husk ash can be converted into mesoporous silica nanoparticles (MSNs), bioactive glasses (BGs), silica–carbon composites and thin films [138,139]. These materials exhibit high surface area, tunable porosity (Figure 3) and versatile surface chemistry, enabling applications in drug delivery, biosensing, wound healing and tissue engineering [62]. Unlike conventional silica synthesis relying on costly tetraethyl orthosilicate (TEOS), the valorization of agricultural waste provides an eco-friendly and economical alternative [62].

In biosensing, mesoporous silica derived from rice husks has been used as a high-surface-area carrier for enzyme immobilization and redox mediator loading. Functionalization with amine or thiol groups allowed covalent coupling of glucose oxidase or horseradish peroxidase, resulting in electrochemical biosensors with lower detection limits and longer operational lifetimes compared to unsupported enzymes [140]. A study from [140], reports that mesoporous silica derived from rice husk ash (RHA) treated with glycerol, with subsequent aging and calcination, yields materials of ~95 weight percent silica and organic residues <3 wt %. The authors loaded ibuprofen and studied release kinetics, demonstrating viability of the material as a drug carrier scaffold [141].

Rice husk silica has also been applied to produce bioactive glasses, which have demonstrated accelerated wound healing in diabetic animal models through enhanced angiogenesis, collagen deposition and controlled release of therapeutic ions [142]. Since bioactive glasses are already clinically approved for use in bone repair and dental products, substituting conventional silica with rice husk ash represents the most clinically feasible route, if purity and compositional reproducibility are guaranteed [143].

Finally, incorporating rice husk silica into polymeric matrices, such as chitosan or cellulose, improves mechanical strength, antimicrobial performance, and supports the osteogenic differentiation of seeded cells in vitro, suggesting its utility in scaffolds and membranes for bone and tissue engineering [140].

When critically compared, bioactive glasses derived from rice husks emerge as the most promising candidates for clinical application, owing to existing regulatory precedents and encouraging preclinical data. Polymeric composites incorporating rice husk silica hold intermediate translational potential for topical devices such as dressings and filtration membranes. By contrast, MSN-based drug delivery systems and Ag-containing composites still face important barriers in terms of safety, reproducibility and regulatory acceptance. Ultimately, successful translation requires standardized purification protocols, validated endotoxin removal, scalable synthesis routes and comprehensive toxicological evaluation (Table 3) [144].

The benchmark for bioactive glass in clinical applications is 45S5 Bioglass^®^ (commercialized under various trade names such as Novabone^®^). For rice husk-derived silica to be commercially viable, it must demonstrate bio-equivalence to this standard. Comparative studies have synthesized bioactive glasses using rice husk ash (RHA) as the silica source and tested them against conventional 45S5. Notably, RHA-derived glasses have demonstrated apatite-forming ability (the marker of bioactivity) in Simulated Body Fluid (SBF) that is kinetically equivalent to commercial 45S5. Moreover, in wound healing models, RHA-based glasses have shown accelerated angiogenic properties compared to standard controls, likely due to the preservation of mesoporous structures during the sol-gel processing often used for waste-derived silica. This suggests that RHA-silica is not merely a low-cost filler but a high-performance alternative capable of replacing mined silica in FDA-approved glass formulations [142].

### 4.3. Calcium Carbonate from Seashells

Marine shells from mollusks and crustaceans, as well as fish bones, are rich natural sources of calcium carbonate (CaCO_3_). When incorporated into scaffold matrices, CaCO_3_ contributes to hemostasis, offers osteoconductive behavior, buffers local pH, and acts as a biodegradable filler in physiological environments [145]. The addition of CaCO_3_ reinforces the mechanical integrity of composite scaffolds and enables controlled release of Ca^2+^ ions which support bone mineralization and remodeling. Specifically, aragonite-form CaCO_3_ nanocrystals extracted from cockle shells have been reported to promote osteoblastic differentiation and improved osteointegration when compared to conventional calcium phosphate fillers [146].

In a pivotal study in 2014, Kamba et al. evaluated cockle-shell-derived CaCO_3_ nanocrystals on hFOB 1.19 and MC3T3-E1 osteoblast lines: they found enhanced cell viability, proliferation, alkaline phosphatase activity, protein synthesis, and extracellular calcium deposition (without significant cytotoxicity), indicating robust osteogenic stimulation [147]. Another work by Mahmood et al. developed a gelatin–aragonite composite scaffold using cockle-shell nanoparticles, characterized its microstructure and mechanical behavior, and implanted it in an animal bone defect, achieving favorable bone formation and scaffold performance [148].

Beyond bone scaffolding, shell-derived CaCO_3_ powders have been adapted as wound fillers: these biomaterials accelerate clot formation, modulate pH microenvironment, and support tissue repair, showing a performance in some models comparable to commercial calcium phosphate wound materials. The dual use (wound filler + scaffold) is discussed in Mailafiya et al. (2019) in the context of cockle-shell aragonite nanoparticles [149].

While promising, this approach raises challenges: natural variability in shell composition may compromise reproducibility; metastability of aragonite may lead to undesirable phase conversion to calcite; balancing mechanical reinforcement with porosity and degradation is nontrivial; sterilization processes risk structural changes; and large-scale production must be cost-effective. Moreover, long-term in vivo studies (especially in large animals or humans) remain limited, and the scale-up from lab to clinic must address consistency, regulatory hurdles, and biological safety [145].

In summary, marine shells and fish-bone-derived CaCO_3_, notably as aragonite nanocrystals, show strong potential as bioactive fillers for composite scaffolds and wound healing formulations. Case studies support their capability to rival or surpass calcium phosphate–based materials in promoting osteogenesis and tissue repair. However, to reach clinical translation, rigorous standardization, phase stability control, extended in vivo validation, and industrial scalability must be achieved (Table 3) [145].

### 4.4. Magnesium from Agro-Industrial Residues

Magnesium extracted from agro-industrial residues is being considered for temporary orthopedic implants due to its biodegradability and osteogenic potential [150]. Magnesium-based alloys exhibit inherent corrosion under physiological conditions, allowing gradual in vivo degradation and thus potentially obviating the need for surgical removal. These alloys also stimulate bone formation, making them promising candidates for orthopaedic devices [151].

Recent studies provide evidence of these potentials. Porous magnesium scaffolds in rabbits promoted osteogenesis over hydroxyapatite controls, though scaffold integrity declined as corrosion progressed. MgYREZr alloy screws implanted for up to 52 weeks in rabbits showed good bone contact and negligible systemic toxicity. Surface treatments, such as PEO and microarc oxidation of Mg-Zn-Ca or AZ31 alloys, notably slow down corrosion, reduce adverse reactions, and improve implant-bone bonding. Reinforcement of magnesium alloys with calcium carbonate (CaCO_3_) powder has shown improvement in mechanical strength, hardness, and corrosion resistance, hinting at synergistic use of residue-derived natural fillers [152].

Nevertheless, several critical challenges remain. Purity of residue-derived magnesium must be ensured to avoid harmful impurities. Control of the degradation rate is essential to maintain mechanical support until bone healing is complete. Mechanical properties must be balanced with biological behavior. Immunological response to Mg degradation must be minimized. Finally, most data come from small animal models; larger and longer-term in vivo studies (and ultimately clinical trials) are needed to verify safety, efficacy, and consistency (Table 3) [153].

### 4.5. Hybrid Systems

Hybrid carriers that integrate polysaccharides, proteins, and inorganic fillers are under intense development to realize multifunctional biomaterials. These systems aim to unite the biocompatibility of polymers, bioactivity of proteins or growth factors, and mechanical reinforcement or functional features of minerals. Examples include cellulose–hydroxyapatite composites for bone-targeted drug release [56], chitosan–silica nanoparticles for pH-responsive anticancer therapy [139,140,141], and pectin–chitosan–HA systems for co-delivery of antibiotics and growth factors in bone infections [73].

Recent work has advanced these ideas. For instance, three-dimensional cellulose–hydroxyapatite scaffolds enriched with dexamethasone-loaded MOFs deliver drug locally and show cytocompatibility and sustained release (≈16% loading, ≈ 60–80 nm MOF particles) [154]. Chitosan/nano-hydroxyapatite scaffolds containing simvastatin-loaded PLGA microspheres exhibit prolonged drug release (~30 days), enhancing osteogenic differentiation in vitro and promoting bone repair in calvarial defect models in rats [155]. Cellulose scaffolds with nano-HA vs. micro-HA showed that nano-HA led to more cell spreading, higher metabolic activity, and elevated osteoblastic gene expression, along with interconnected porosity suitable for bone ingrowth [56]. On the anticancer side, chitosan-coated mesoporous silica systems loaded with curcumin or olaparib show pH-sensitive release (more release under acidic conditions) and improved cytotoxicity in cancer cell lines vs. free drug [156].

However, these hybrid systems also come with challenges: reproducibility, balancing mechanical vs. biological properties, controlling initial burst release, ensuring targeting and stimuli responsiveness in vivo, verifying biocompatibility and safe degradation, and controlling cost and regulatory complexity (Table 3).

### 4.6. Critical Comparative Analysis

In the realm of inorganic fillers, microstructure and ionic composition drive performance differences. Hydroxyapatite (HA) derived from eggshells exhibits a nanocrystalline structure that closely resembles the mineral phase of human bone. Its primary advantage lies in cellular interactions; the presence of trace ions (Mg, Sr) in the crystal lattice has been shown to upregulate osteogenic gene expression more effectively than stoichiometric synthetic HA [132]. In terms of mechanical properties, however, HA is brittle and serves best under compressive loads. Rice husk-derived silica (bioactive glass), on the other hand, functions through a different mechanism: its amorphous microstructure and high surface area allow for rapid ion exchange, releasing silicic acid that stimulates angiogenesis [143]. While HA provides a stable osteoconductive scaffold, silica-based glasses are more bioactive and degradable, making them better suited for stimulating soft tissue repair or rapid bone turnover rather than permanent structural support.

### 4.7. Quantitative Performance Benchmarks

To enable an objective comparison between agro-waste-derived hybrids and conventional biomaterials, it is essential to analyze specific numerical indicators regarding purity, drug release kinetics, and degradation profiles. Recent literature provides concrete metrics that validate the competitive performance of these sustainable systems.

The clinical viability of waste-derived inorganics is often questioned regarding impurity levels; however, quantitative analysis has demonstrated that optimized extraction protocols can achieve high-grade materials. For instance, mesoporous silica nanoparticles (MSNs) synthesized from rice husk ash (RHA) via glycerol treatment and calcination have yielded silica purity levels of ~95 weight percent, with residual organic content reduced to <3 weight percent. This purity profile is critical, as it falls within the acceptable range for biomedical carriers, comparable to commercial silica sources used in drug delivery [141]. In terms of therapeutic performance, hybrid scaffolds have demonstrated quantifiable drug loading and sustained release capabilities. Cellulose–hydroxyapatite nanocomposites functionalized with metal-organic frameworks (MOFs) achieved a drug loading capacity of approximately 16% using MOF particles in the 60–80 nm size range [154]. This high surface-area-to-volume ratio facilitated the sustained local delivery of dexamethasone. Similarly, chitosan/nano-hydroxyapatite scaffolds designed for bone repair demonstrated a prolonged release profile of simvastatin extending over 30 days [155]. This specific timeframe is numerically significant as it aligns with the early inflammatory and osteogenic phases of bone healing, offering a distinct advantage over systems that suffer from rapid “burst release” within the first 24 h. Objective data regarding in vivo longevity is also available for metallic hybrids. Magnesium-based alloy implants (e.g., MgYREZr) derived from recovery processes have been shown to maintain mechanical integrity and bone contact for up to 52 weeks in rabbit models. This specific duration indicates that these materials possess a degradation rate slow enough to support osteogenesis during the critical remodeling period, yet fast enough to eventually clear from the system, confirming their utility as temporary load-bearing devices [152].

These numerical indicators collectively confirm that agro-waste-derived materials are not merely conceptually appealing but possess the quantifiable physicochemical properties required for clinical translation.

## 5. Phytochemical-Rich Extracts from Fruit Residues in Biomaterials Development

Fruit processing residues represent a sustainable source of phytochemicals, including polyphenols, flavonoids, and other bioactive compounds, which can be harnessed for biomaterials development. These natural extracts not only provide antioxidant, antimicrobial, and anti-inflammatory functions but also enhance the biocompatibility and functionality of polymers and composites [8,157]. By integrating phytochemical-rich extracts into biomaterial design, it is possible to create innovative systems for wound healing, tissue engineering, and drug delivery. Moreover, their use supports the principles of circular bioeconomy, transforming fruit byproducts into high-value resources for biomedical applications [6,7,157].

Fruit pomace and peel residues are rich in bioactive compounds, such as polyphenols, flavonoids, and tannins, which act as antioxidants, antibacterials, and biofilm inhibitors [158]. Pomegranate peel extract (PPE) incorporated into hydrogels has been shown to accelerate wound healing in diabetic models by modulating collagen deposition and reducing oxidative stress [159,160].

Hybrid systems combining PPE with silver nanoparticles and hyaluronic acid exhibit both antifungal and regenerative effects in wounds infected with *Candida albicans* [161]. Similarly, grape pomace extracts integrated into chitosan/alginate hydrogels enhance swelling, antioxidant protection, and antibacterial performance, promoting rapid re-epithelialization [162,163].

**Figure 4:**
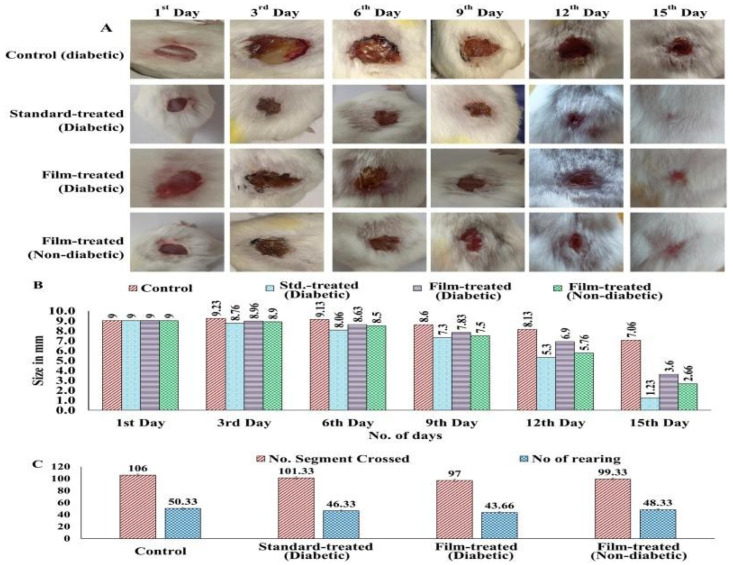
(**A**) Photographs of wound healing of diabetic and mice treated with standard and optimized films and (**B**) graphical representation of wound closer area over 15 days. (**C**) Assessment of locomotor activity of mice after wound healing by open field test Adapted with permission from Savekar et al. [160].

Recent studies extend these insights. For example, PPE formulated as methanolic extract gel improved diabetic rat wound healing, increasing hydroxyproline, factors such as VEGF, EGF, and TGF-β1, and reducing oxidative stress while accelerating closure time. Hydrogel films composed of citric acid cross-linked β-cyclodextrin/carboxymethylated tapioca starch incorporating PPE release ellagic acid in a pH-responsive manner and promote epithelialization and angiogenesis in diabetic mouse models (Figure 4) [164]. Grape pomace extracts embedded in chitosan/alginate hydrogel matrices display improved antimicrobial activity (including against *S. aureus*), better swelling and exudate handling, features beneficial for wound dressings. Moreover, coatings and functionalization of hydroxyapatite/chitosan composites with grape pomace polyphenols enable smart release in inflammatory conditions and retain antioxidant activity over time [85,162].

Nevertheless, challenges remain. Standardizing extract composition, ensuring stability and controlled release, verifying cytocompatibility and immune safety, especially when co-agents (like silver or nanoparticles) are used, balancing mechanical, swelling, and degradation properties, translating from small animal models to clinical contexts, and addressing cost, regulation, and scalable manufacture (Table 4) [85,158,162].

### Hybrid Systems

The integration of phytochemical-rich extracts into nanocellulose, pectin, or chitosan matrices, reinforced with inorganic phases such as rice husk-derived silica or eggshell calcium phosphate, has led to multifunctional dressings that combine moisture regulation, mechanical reinforcement, antioxidant capacity, and antimicrobial ion release [42]. Polyphenols, tannins, and flavonoids from agricultural residues can act not only as therapeutic agents but also as reducing and stabilizing agents for the green synthesis of metallic nanoparticles such as silver or zinc oxide, which when incorporated into polymeric matrices enhance antimicrobial efficacy against multidrug-resistant pathogens [24,61,84,165]. For instance, sugarcane bagasse-derived nanocellulose scaffolds enriched with polyphenols improved wound closure and epithelialization in diabetic rat models, while chitosan-silver nanocomposites synthesized using *Saccharum officinarum* extracts showed sustained antifungal and antibacterial effects in vitro and controlled itraconazole release for chronic wounds [166,167]. Similarly, bacterial cellulose nanopaper impregnated with in situ reduced silver nanoparticles demonstrated transparency and responsiveness for colorimetric detection of toxins, highlighting the potential of these systems to serve as both therapeutic dressings and biosensors [168]. Other approaches, such as embedding silver nanoparticles synthesized via *Camellia sinensis* leaf extracts into chitosan gels, revealed significant inhibition of *Escherichia coli* and *Staphylococcus aureus* while also providing antioxidant activity, illustrating the synergistic effect of phytochemicals and metallic nanoparticles in hybrid composites [169]. Despite these promising outcomes, challenges remain. Extract variability due to plant origin, harvest, and processing conditions complicates reproducibility; nanoparticle dosage must be tightly controlled to avoid cytotoxicity or induction of microbial resistance; and mechanical stability can be compromised when high extract or nanoparticle loading alters swelling or degradation behaviour [170]. Moreover, the release kinetics of phytochemicals and nanoparticles require fine-tuning to balance rapid antimicrobial action with long-term stability, and regulatory translation is limited by the need to comply with ISO 10993 standards for cytocompatibility and to control nanoparticle leaching [171]. Finally, although life cycle analyses suggest that agricultural waste-derived fillers like rice husk silica or eggshell calcium carbonate have lower environmental footprints compared to mined materials, energy-intensive purification and sterilization still raise sustainability questions [172]. Collectively, these studies underscore the potential of hybrid systems based on agro-waste-derived polysaccharides, phytochemicals, and inorganic fillers, while highlighting the urgent need for standardized protocols, comprehensive toxicological assessments, and scalable green manufacturing routes before clinical adoption becomes feasible (Table 4).

## 6. Sustainability Aspects

Efficient and green extraction and purification of bioactive compounds and inorganic materials from agricultural residues are essential to unlocking the potential of waste-derived biomaterials in biomedical applications. Conventional methods, such as solvent extraction using high concentrations of organic solvents and high-temperature treatments, frequently demand large energy inputs, generate chemical waste, and may degrade sensitive bioactive molecules, thereby limiting their sustainability and scalability. Alternative approaches—including enzymatic delignification and hydrolysis (using cellulases, hemicellulases, laccases) to selectively degrade non-cellulosic fractions—allow access to nanocellulose or other polysaccharides under milder conditions, reducing environmental impact. Mechanochemical pretreatments (ball-milling combined with mild chemical activation) help lower biomass recalcitrance, while microwave- or ultrasound-assisted extraction can accelerate mass transfer, reduce solvent volumes and duration, and often preserve phytochemical integrity [173].

Several concrete case studies illustrate these green approaches. One study compared microwave- versus ultrasound-assisted extraction from olive pomace and by-products: microwave treatment gave higher yields of phenolic compounds (especially oleuropein and hydroxytyrosol), while ultrasound was favorable for selectivity in mild conditions. Mild drying versus untreated samples was also compared, with untreated material giving higher total yields but dried material showing higher phenolic purity [174]. In another example, olive leaf extraction by microwave-assisted extraction (MAE) of various Italian cultivars under different harvest times, solvents, and power/time settings revealed that cultivar and phenological time (harvest time) had greater influence on phenolic/flavonoid content than extraction time or microwave power. Ethanol-water mixtures were particularly effective [175]. For polysaccharides, sugarcane bagasse has been used to extract nanocellulose whiskers via acid hydrolysis (60% sulfuric acid) at moderate temperature (≈45 °C), obtaining needle-like cellulose whiskers whose crystallinity improved with hydrolysis time, though yield dropped at longer hydrolysis due to over-degradation of amorphous cellulose [176].

For inorganic materials, such as silica from rice husk or calcium carbonate from eggshells, green strategies are also under investigation. Acid leaching followed by alkaline extraction and sol-gel conversion, sometimes assisted by microwave heating, has been used for silica extraction to produce high-purity amorphous silica, reducing process time and energy consumption [177]. Eggshell hydroxyapatite synthesis may combine precipitation or hydrothermal routes with bio-templating additives—such as plant extracts—to control crystallinity, morphology, and particle size; such templating can help reduce use of harsh chemicals or reduce synthesis temperature. Co-locating extraction facilities near agro-industrial sites (near mills, farms, juice or olive oil plants) can further reduce transport energy and emissions, facilitating circular material flows [178].

Critical sustainability aspects must be considered when evaluating these methods. First, energy vs. yield trade-offs are recurring. While microwave or ultrasound methods reduce extraction time, they often require specialized equipment and may incur high energy per unit of biomass, and their benefit depends on optimizing parameters (solvent, power, time) [179]. Moreover, solvent choice and chemical inputs matter: using water or low percentages of green solvents (e.g., ethanol, deep eutectic solvents) tends to be preferable to harsh chemicals but sometimes yield or purity suffers. Additionally, extract stability and compound integrity can be compromised under high temperature, prolonged exposure, or severe chemical conditions, reducing antioxidant or bioactive potency [180].

Along these lines, purity and safety are important: residues of solvents, heavy metals (especially when extracting silica, or if biomass was contaminated), or residual enzymes or chemical activators must be removed to meet biomedical standards [181]. Scalability and process economics can also limit translation. Techniques that work in small lab-scale batches (e.g., enzymatic hydrolysis, ultrasound extraction) may face challenges when scaling up due to cost of equipment, process control, and consistency of feedstock. The variability of biomass (moisture, composition) poses additional challenges in maintaining reproducibility [182]. Life cycle and environmental impacts need consideration as the total carbon footprint, water usage, waste streams, and possible environmental hazards of purification steps (e.g., acid/base neutralization) must be evaluated. Even techniques with low chemical usage sometimes require post-treatment to treat waste [183].

Finally, regulatory compliance and safety must be front of mind. For biomedical applications, extracts and materials must satisfy standards for cytocompatibility (e.g., ISO 10993), ensure removal of toxic residues, verify that any inorganic material (e.g., silica, hydroxyapatite) meets purity and crystallographic specifications, and that bioactive molecule extraction does not introduce unwanted immunogenic or mutagenic substances [171].

In sum, while green extraction and purification routes from agro-waste offer strong promise in terms of sustainability, functionality, and circular economy benefits, realizing their potential demands careful optimization of extraction parameters, solvent and energy use, biomass preprocessing, purity control, and scale-up, along with rigorous safety and regulatory validation.

### 6.1. Surface Chemistry and Bioconjugation

When biomaterials are prepared (polysaccharides, silica, calcium phosphate from agro waste), tuning their surface chemistry becomes crucial to ensure proper interaction with biomolecules or cells. One common strategy employs carbodiimide chemistry (EDC/NHS) to create amide linkages between carboxyl groups in polysaccharides or proteins and amine-bearing ligands [184]. In parallel, silica surfaces (such as those derived from rice husk ash) can be modified via silanization (using amine-or epoxy-functional silanes) to graft functional moieties, enhancing enzyme or antibody immobilization. For example, rice husk silica functionalized with 3-aminopropyl triethoxysilane (APTES) has been embedded into chitosan/alginate scaffolds to produce a composite that supports immobilization of laccase, yielding a reusable biocatalyst with good activity and stability under mild conditions [185].

In the polysaccharide domain, periodate oxidation of cellulose or chitosan is regularly used to generate aldehyde groups; these can then react via Schiff base formation with amino-containing biomolecules (peptides, proteins) to form stable conjugates. Notably, a “resource-saving” method combined a brief ball-milling pretreatment with periodate oxidation at high pulp consistency (cellulose/water ≈ 1:4) to yield dialdehyde cellulose (DAC) with high aldehyde content, while minimizing water usage and energy input [185]. Moreover, studies such as periodate oxidation followed by sodium borohydride reduction in microfibrillated cellulose have produced nanocrystals featuring a crystalline core and flexible polyol chains; these were more colloidally stable and have potential as carriers or scaffolds for further conjugation [186].

Layer-by-layer (LbL) assembly of alternating charged biopolymers (for example, chitosan and alginate) offers another modular route to build coatings enriched with biomolecules. An example is LbL films of modified chitosan/alginate matrices serving as scaffolds for human adipose-derived stem cell aggregates, where chitosan derivatives (mesyl/tosyl modifications) allowed tuning of physico-chemical properties which influenced biological performance [187].

These surface functionalization methods enable the embedding of enzymes, antibodies, aptamers, peptides, or growth factors onto materials derived from agro-waste substrates, yielding biosensor elements or bioactive implant surfaces. However, several critical issues emerge in the literature. First, surface density, orientation, and steric accessibility of the immobilized biomolecules significantly affect biological performance: too dense grafting can lead to steric hindrance, loss of activity, or poor diffusion; too sparse grafting can reduce efficiency or signal. Furthermore, the generation of aldehyde or amine groups often requires oxidation, silanization or chemical activation steps that may damage the substrate (e.g., reduce mechanical strength, alter porosity, degrade sensitive functional groups). For instance, periodate oxidation of crystalline cellulose decreases crystallinity and may reduce tensile strength or alter its morphology [188].

Moreover, the stability of the conjugates under physiological conditions (pH, ionic strength, presence of enzymes) must be validated. Schiff base linkages (aldehyde-amine) are reversible in some conditions unless reduced or otherwise stabilized; silane grafts may hydrolyze in aqueous environments if bonding is imperfect [189]. In addition, reproducibility and batch variation are nontrivial: source of agro-waste material (rice husk, cellulose feedstock) may vary in purity, impurity levels (e.g., residual metals in silica), and surface group density [190]. Biocompatibility and immunogenicity are essential as well. Surface chemistry modifications can lead to unintended reactivity, cytotoxicity, or triggering of immune responses if non-native chemistries or residual reagents (e.g., unreacted silanes, residual periodate, incomplete washing) are present [191].

Case studies also highlight scale and reuse considerations: the rice husk silica/chitosan/alginate composite with APTES functionalization (ARCA composite) preserved about 50% of laccase activity after six reuse cycles, indicating promise for reusability while demonstrating that repeated use imposes stress on conjugated systems [185]. Another case: soybean lipoxygenase immobilized on nanoporous rice husk silica by adsorption (without covalent coupling) achieved ~50% adsorption efficiency, and the immobilized enzyme retained 73% of soluble enzyme activity under high substrate concentration, although efficiency dropped for low substrate levels and some desorption occurred [192].

In summary, surface chemistry and bioconjugation strategies are fundamental to exploiting agro-waste-derived biomaterials for sensors, implants, and drug delivery. Success depends on optimizing functionalization method, balancing stability vs. activity, ensuring material integrity, rigorous purification and consistency, and verifying biocompatibility (Table 5).

### 6.2. Composite Formation and Additive Manufacturing

To overcome limitations in mechanical strength, electrical conductivity, or processability, waste-derived biomaterials are often blended into composites. Polysaccharides or cellulose from agro-residues can be combined with biodegradable polymers (e.g., PLA, PCL) or conductive fillers (e.g., graphene, carbon black) to tailor mechanical and electrochemical behavior. For instance, eggshell-derived calcium carbonate (or converted HA) has been used as a filler in PLA composites, enhancing stiffness while lowering cost [193]. In the hydroxyapatite realm, doping with Zn or Ag ions has been shown to enhance interfacial adhesion between HA and polymers (e.g., PCL) in composite scaffolds, improving mechanical integrity and antimicrobial properties [194].

Additive manufacturing approaches are increasingly applied to these composites. Direct ink writing or extrusion-based 3D printing has been used to fabricate porous scaffolds from cellulose-rich inks and eggshell-derived HA formulations with controlled architecture [195]. One illustrative example is the fabrication of eggshell-based osteoinductive multiphasic calcium phosphate scaffolds using a coagulation-assisted extrusion and sintering route, which yielded hierarchical porosity and improved mechanical performance compared to conventional apatite scaffolds [196].

Electrospinning of straw- or corn-stalk-derived cellulose into nanofibrous membranes has also been achieved, pro ducing extracellular matrix–mimicking architectures useful in both sensors and implantable devices.

### 6.3. Obstacles, Limitations, and Regulatory Aspects

While the potential of agro-waste biomaterials is vast, the technological barriers preventing their translation from the laboratory to the clinic are substantial and often underestimated. Unlike synthetic biomaterials produced in controlled environments, agricultural residues are subject to intrinsic biological variability and extrinsic contamination risks that pose severe challenges for standardization and safety [197,198].

A central challenge in leveraging agro-waste is batch-to-batch heterogeneity. Agricultural residues are biological products influenced by genetic factors (cultivar differences), environmental conditions (soil composition, climate, rainfall), and harvest timing. These variables result in significant fluctuations in the physicochemical properties of the derived materials, such as the degree of acetylation in chitosan, the crystallinity index of cellulose, or the molecular weight distribution of proteins. Without standardized quality control protocols—which are currently lacking for waste-derived streams—it is difficult to guarantee the reproducible mechanical strength and degradation rates required for medical devices [199].

Perhaps the most critical barrier is the “chemical memory” of the waste feedstock. Agricultural raw materials are frequently exposed to agrochemicals and environmental pollutants. Consequently, residues may contain pesticides and herbicides. Trace residues of crop protection agents can be co-extracted with the biomass. Even at low concentrations, these compounds may exhibit cytotoxicity or endocrine-disrupting effects incompatible with ISO 10993 biocompatibility standards [171]. Additionally, storage of agricultural waste often promotes fungal and bacterial growth. This introduces the risk of mycotoxins (e.g., aflatoxins) and bacterial endotoxins (lipopolysaccharides). Endotoxins, in particular, are difficult to remove as they are heat-stable, and their presence on implant surfaces can trigger severe immunogenic reactions or septic shock. Moreover, plants such as rice and leafy crops can bioaccumulate heavy metals (e.g., arsenic, lead, cadmium) from the soil. When processing large volumes of biomass to extract silica or cellulose, these metals can become concentrated in the final biomaterial [200,201].

Purification protocols must therefore be rigorous, often requiring multi-step processes (e.g., chelation, dialysis, solvent washing) that may paradoxically reduce the “green” credentials of the material or degrade its bioactive components.

Achieving the Sterility Assurance Level (SAL) required for implantable devices (10^−6^) is particularly challenging for natural polymers. Standard sterilization methods often damage the structural integrity of agro-waste derivatives. Indeed, in the thermal sterilization (autoclaving), high temperatures can cause hydrolysis of polysaccharide chains and denaturation of proteins like collagen or keratin, leading to a loss of mechanical properties. Gamma irradiation, while effective, often causes chain scission in cellulose and chitosan, reducing tensile strength and altering degradation profiles. Ethylene Oxide (EtO) is less damaging to structure, but requires the material to be permeable to gas and free of toxic residues after aeration, which can be difficult to ensure in dense nanocomposites [202].

To address these issues, the development of these biomaterials must move beyond simple characterization. Advanced analytical control is required, including High-Performance Liquid Chromatography (HPLC) and Mass Spectrometry (MS) to detect trace pesticide residues, and Limulus Amebocyte Lysate (LAL) assays to quantify endotoxin levels. Developing standardized “Master Files” for waste-derived feedstocks that characterize these impurity profiles is an essential step toward regulatory approval that the field has yet to fully embrace [201,202].

Early engagement with regulatory agencies and use of standardized preclinical models (e.g., mechanical fatigue, biofilm assays, immunogenicity testing) can facilitate the pathway to human use (Table 5) [201,202].

### 6.4. Future Directions and Research Priorities

Future research on agro-waste-derived biomaterials should prioritize the establishment of open-access databases that systematically connect physicochemical parameters—such as the degree of esterification in pectin, crystallinity index of cellulose, or trace-metal content in biosilica—with functional performance in biomedical devices, enabling data-driven material optimization rather than empirical trial-and-error. At the same time, environmentally friendly processing routes including enzymatic delignification, microwave- or ultrasound-assisted extraction, and microbial synthesis of nanostructures are emerging as scalable, low-footprint alternatives to conventional chemical treatments [66]. Promising case studies illustrate how such strategies can be leveraged: eggshell-derived hydroxyapatite not only serves as a low-cost substitute for synthetic HA but, when engineered as composite coatings, can deliver osteoinductive and antimicrobial functions [178]; rice husk silica has been employed in implant surface modifications to improve corrosion resistance and osseointegration [88,144,177]; and banana peel-derived nanocellulose films are being explored for flexible biosensors with antibacterial properties [203]. The next frontier lies in multifunctional integration—such as embedding nanoscale sensors within HA coatings to monitor osseointegration in situ or developing conductive nanocellulose-based films that combine antimicrobial release with real-time biosensing for wearable platforms. Critical to clinical translation will be robust validation of sterilization compatibility, long-term in vivo performance across multiple models, and structured roadmaps to pilot human trials. Yet, despite encouraging preclinical advances, a translational bottleneck persists only a handful of agro-waste biomaterials have reached clinical testing, reflecting the fragmented regulatory landscape and the underestimation of scale-up challenges [41]. Therefore, research priorities should not only target novel functionalities but also emphasize harmonized standards, reproducibility, and regulatory dialogue to ensure that sustainability-driven innovations evolve from laboratory prototypes into clinically viable technologies (Table 5) [199,200,201,202].

## 7. Conclusions

Agricultural and agro-industrial residues represent a vast and largely untapped resource for the development of sustainable biomedical materials. Their chemical diversity—spanning polysaccharides, proteins, inorganic minerals, and phytochemicals—enables the fabrication of multifunctional systems that combine biocompatibility, biodegradability, and tunable bioactivity. Recent progress demonstrated that waste-derived biomaterials can rival or even surpass conventional synthetic counterparts in applications such as tissue engineering, wound healing, drug delivery, and biosensing.

Among the explored materials, polysaccharide-based hydrogels and nanocellulose composites stand out for their advanced translational readiness, particularly in wound care and bone regeneration. Protein-based scaffolds derived from collagen, gelatin, keratin, and soy protein also offer promising alternatives for regenerative medicine, while inorganic residues such as eggshell-derived hydroxyapatite and rice husk silica provide sustainable routes to bone and dental materials. The integration of these biogenic components into hybrid systems further expands their potential by combining biological functionality with mechanical robustness and controlled therapeutic delivery.

Despite these advances, key challenges remain—namely, ensuring reproducible extraction and processing, maintaining consistent physicochemical quality, scaling up under green and economically viable conditions, and navigating complex regulatory frameworks. Addressing these issues through interdisciplinary collaboration among chemists, materials scientists, and biomedical engineers will be essential for successful clinical translation.

Overall, the valorization of agro-residues exemplifies a paradigm shift toward a circular bioeconomy, where waste streams are transformed into high-value biomedical resources. By aligning ecological stewardship with medical innovation, these next-generation biomaterials can simultaneously promote human health and environmental sustainability.

## Figures and Tables

**Table 1:** Overview of polysaccharide-based biomaterials derived from agro-industrial waste, their biomedical applications, representative case studies, key material advantages, and relative technological readiness.

Polysaccharide Source	Application Domain	Representative Examples	Key Advantages	Readiness Status
Cellulose nanofibrils (CNFs)	Bone regeneration and issue engineering	CNF–hydroxyapatite (HA) composites; mineralized scaffolds mimicking ECM and supporting osteogenic differentiation	Mechanical reinforcement + osteoconductivity; ECM-like nanoscale structure	Most advanced
Cellulose nanocrystals (CNCs)	Drug delivery	CNC nanoparticles functionalized for pH-responsive 5-FU release in colon cancer	High surface area; tunable surface chemistry; controlled release	Preclinical
Nanocellulose (CNFs/hydrogels/aerogels)	Wound healing/dressings	Nanocellulose hydrogels and aerogels; rice husk-derived nanocellulose/ZnO antimicrobial composites	Moisture retention; exudate absorption; antimicrobial functionalization possible	Near clinical
Cellulose nanostructures (CNFs/CNCs)	Biosensors/wearable devices	CNFs + graphene for enzyme immobilization; electrospun cellulose fibers for epidermal sensors monitoring sweat metabolites	Flexible, porous matrix; supports conductive additives; high sensitivity	Intermediate maturity
Nanocellulose hydrogels	Soft tissue engineering/temporary implants	Hydrogels for cartilage repair and local drug depots	High water content; biocompatibility; tunable degradation	Early stage
Pectin	Wound healing	Citrus/passion fruit pectin hydrogels and pectin-chitosan films; bioadhesive, antimicrobial	Intrinsic antimicrobial/antioxidant activity due to co-extracted phytochemicals	Most mature
Pectin	Drug delivery	Colon-targeted 5-FU microspheres; oral insulin and oral vaccine delivery nanoparticles	Microbiota-triggered release; improved mucosal absorption	Preclinical
Pectin	Tissue engineering (cartilage)	Pectin–chitosan polyelectrolyte complexes	Improved stability and cell adhesion	Early stage
Chitosan	Drug delivery (oral peptides)	Nanoparticles from mushroom residues for oral insulin	Mucoadhesive; enhances intestinal permeability	Preclinical
Chitosan	Drug delivery (ocular)	Chitosan-based ophthalmic antibiotic systems	Increased residence time; reduces dosing frequency	Near clinical
Chitosan	Gene/nucleic acid delivery	Chitosan–DNA and chitosan–siRNA complexes	Non-viral, biodegradable delivery vector	Early stage
Chitosan	Wound healing	Chitosan + cellulose + ZnO antimicrobial dressings; chitosan + polyphenols	Hemostatic; antimicrobial; promotes angiogenesis	Most mature
Chitosan hydrogels	Soft tissue filler/temporary implant	Injectable hydrogels for cartilage regeneration and local drug release	Biocompatible; tunable degradation	Intermediate maturity
Hybrid systems (polysaccharide blends/composites)	3D bioprinting & bioinks	Pectin–chitosan bioinks; nanocellulose + alginate/gelatin bioinks	Printability; shape fidelity; cell compatibility	Near clinical
Hybrid systems (polysaccharide–inorganic)	Bone/dental regeneration; theranostics	Cellulose–silica composites; chitosan/dextran matrices with metallic nanoparticles (Au, Fe_3_O_4_, silica)	Mechanical reinforcement; stimuli-responsive behavior; drug delivery + imaging	Varies

**Table 2:** Summary of protein-based biomaterials obtained from agro-industrial residues, including application domains, representative examples, functional advantages, and current translational status.

Protein Source	Application Domain	Examples	Key Advantages	Readiness Status
Collagen	Wound healing and advanced dressings	Electrospun collagen nanofibers; Eggshell membrane (ESM) dressings	Mimics ECM, promotes angiogenesis and re-epithelialization; strong antibacterial effect when loaded	Clinical/commercial
Collagen	Bone regeneration	Collagen–hydroxyapatite composites for bone grafts and dental applications	Osteoconductive, integrates into host bone	Clinical practice
Collagen	Drug delivery	Collagen hydrogels and sponges loaded with antibiotics or growth factors	Sustained release; bioactive scaffold	Translational/preclinical
Gelatin	Drug delivery (nanoparticles, microspheres)	Gelatin nanoparticles loaded with doxorubicin; gelatin microspheres delivering BMPs	Easy to functionalize, controlled release, tumor targeting	Preclinical
Gelatin (GelMA)	3D bioprinting/cartilage & cardiac tissue engineering	GelMA injectable hydrogels/bioinks used for in situ photopolymerization	Tunable stiffness, printability, supports angiogenesis	Near clinical trials/large animal studies
Gelatin	Wound healing	Gelatin nanofibers loaded with ciprofloxacin	Accelerated wound closure, antibacterial	Preclinical
Keratin	Wound healing/dressings	Keratin/polyphenol composite sponges; keratin–silver nanocomposites	Intrinsic antimicrobial + antioxidant activity; moisture retention	Closest to clinical among keratin systems
Keratin	Drug delivery (nanoparticles)	Keratin nanoparticles carrying paclitaxel; folate-functionalized keratin nanoparticles for targeted delivery	Amphiphilic; controlled drug release	Preclinical
Keratin	Nerve regeneration	Keratin conduits	Slow degradation, nerve growth support	Early translational; animal studies
Soy protein	Soft tissue engineering and scaffolds	Electrospun soy nanofibers; porous sponges	Plant-based, low cost, bioactive peptides promote adhesion	Preclinical
Soy protein	Wound healing	Soy hydrogels blended with polysaccharides	Reduced zoonotic risk, enhances angiogenesis and granulation	Closest to application among soy systems
Soy protein	Drug delivery	Soy nanoparticles delivering curcumin or doxorubicin	Improves solubility & bioavailability; sustained release	Preclinical
Hybrid systems (protein + hydroxyapatite)	Bone regeneration	Keratin–hydroxyapatite scaffolds; alternative to collagen–HA	Combines keratin’s bioactivity with HA osteoconductivity	Preclinical
Hybrid systems (protein + cellulose nanofibers)	Soft tissue and wound healing	Soy protein–cellulose nanofiber composites	Stronger mechanical properties, reduced water solubility	Preclinical
Hybrid systems (protein + bacterial cellulose)	Wound healing and soft tissue repair	Bacterial cellulose–gelatin or cellulose–silk fibroin dressings	Excellent mechanical robustness + bioactivity	Closest to clinical among hybrid systems

**Table 3:** Inorganic biomaterials sourced from agricultural and marine residues, with examples of biomedical applications, key physicochemical benefits, and technological readiness levels.

Inorganic Phase (Source)	Application Domain	Examples	Key Advantages	Readiness Status
Hydroxyapatite (HA) (eggshells and fish bones)	Bone regeneration; bone fillers and cements	Porous HA scaffolds from eggshell calcination supporting bone deposition; injectable ES-HA cements delivering gentamicin or vancomycin for osteomyelitis	Osteoconductive; mimics natural bone mineral; drug-delivery capability; trace ions improve bioactivity	Near clinical
Hydroxyapatite (nano-HA) (eggshell)	Dental remineralization; enamel repair and dentin hypersensitivity	ES-HA toothpastes and gels remineralizing bleached enamel; tubule occlusion	Remineralization; increased enamel microhardness; cost-effective circular alternative	Closest to clinical translation
Hydroxyapatite (fish bone)	Drug delivery and osteoporosis therapy	Fish-HA scaffolds as carriers for bisphosphonates; ion-doped HAs (Mg^2+^, Sr^2+^) enhance osteogenesis	Drug-delivery reservoir; tunable through ion doping	Preclinical
Silica (rice husks)	Drug delivery (MSNs)	Mesoporous silica nanoparticles absorbing ibuprofen; enzyme immobilization for biosensing	High surface area; tunable porosity; eco-friendly alternative to TEOS	Early stage
Bioactive glass (silica-based) (rice husk ash)	Wound healing and bone repair	Rice-husk bioactive glass improving angiogenesis and collagen deposition in diabetic models	Cinically established materials; waste-derived silica reduces cost	Most promising to clinic
Silica–polymer composites (rice husk silica + chitosan/cellulose)	Scaffolds and membranes for bone and tissue engineering	Rice husk silica–chitosan scaffolds supporting osteogenic differentiation	Mechanical reinforcement + antimicrobial effect	Intermediate maturity
Calcium carbonate (CaCO_3_, aragonite) (seashells/cockle shells)	Bone scaffolds and temporary fillers	Gelatin–aragonite scaffolds implanted in bone defect models; osteogenic stimulation in vitro	Hemostatic; osteoconductive; gradual biodegradation	Preclinical
Calcium carbonate powders	Wound healing (clot accelerator)	Cockle-shell powders as wound fillers to accelerate clot formation	Hemostasis + pH buffering; dual scaffold/wound use	Intermediate
Magnesium (from agro-industrial residues)	Biodegradable orthopedic implants (screws, scaffolds)	Mg-based alloys (MgYREZr, Mg-Zn-Ca) showing osteogenesis; corrosion-controlled implants	Biodegradable metal; osteogenic	Emerging
Hybrid systems (inorganic + polymer/protein)	Multifunctional drug-delivery scaffolds and 3D printing	Cellulose–HA scaffolds with MOFs (drug-loaded); chitosan–silica pH-responsive anticancer carriers; pectin–chitosan–HA co-delivery systems	Combines mechanics, bioactivity, controlled release	Varies

**Table 4:** Phytochemical-rich extracts from fruit-processing residues used in biomaterial design, highlighting application areas, main therapeutic functions, and maturity of development.

Phytochemical Source	Application Domain	Examples	Key Advantages	Readiness Status
Pomegranate peel extract (PPE) (polyphenols, tannins, flavonoids)	Wound healing (particularly diabetic wounds)	PPE incorporated into hydrogels accelerates healing by modulating collagen deposition and reducing oxidative stress; PPE methanolic gel release ellagic acid pH-responsively	Antioxidant, antimicrobial, anti-inflammatory; promoted collagen deposition, angiogenesis, epithelialization	High preclinical maturity
PPE hybrid systems (PPE + AgNPs/HA/polysaccharides)	Antifungal wound dressings and regenerative scaffolds	PPE + silver nanoparticles + hyaluronic acid hydrogels show antifungal + regenerative effects; HA/chitosan composites functionalized with PPE for smart release under inflammation	Dual-function: antimicrobial + regenerative; responsive release	Emerging
Grape pomace extracts (rich in polyphenols, anthocyanins)	Wound dressings/re-epithelialization/antibacterial & antioxidant	Grape pomace extracts in chitosan/alginate hydrogels improve swelling, exudate management, antimicrobial activity (incl. *S. aureus*), and accelerate re-epithelialization	Antioxidant, antibacterial, biofilm inhibition; enhances hydrogel hydration and swelling	Intermediate–high
Grape pomace + inorganic composites	Smart release systems (inflammatory-triggered drug delivery)	Hydroxyapatite/chitosan + grape pomace polyphenols enable smart release and maintain antioxidant activity	Multi-functionality: drug delivery, antioxidant, osteo-support	Early preclinical
Bagasse-derived nanocellulose + polyphenols	Diabetic wound healing and tissue repair	Nanocellulose scaffolds enriched with polyphenols improve wound closure and epithelialization in diabetic rat models	Mechanical reinforcement + antioxidant effect	Preclinical
Polyphenol-assisted synthesis of metallic nanoparticles (fruit pomace, Saccharum officinarum, *Camellia sinensis*)	Hybrid antimicrobial/antifungal systems	Green synthesis of AgNPs or ZnO NPs using fruit extracts; AgNP–chitosan composites with antifungal and antibacterial effect and controlled itraconazole release; cellulose nanopaper + AgNPs for colorimetric sensing	Phytochemicals as reducing/stabilizing agents; strong antimicrobial activity; enables biosensing	Early–intermediate
General fruit waste polyphenols & tannins	Biomaterial functionalization and surface coating	Polyphenols used to functionalize polymer and inorganic surfaces; improve biocompatibility and oxidative stability	Improves biocompatibility, antioxidant protection, and adhesion	Intermediate

**Table 5:** Sustainability aspects of agro-waste-derived biomaterials.

Process Area	Key Methods	Materials Derived	Main Advantages	Limitations
Extraction and purification of bioactive compounds and inorganic materials	Enzymatic delignification/hydrolysis (cellulases, laccases); mechanochemical pretreatment (ball-milling); microwave-/Ultrasound-assisted extraction (MAE/UAE); acid hydrolysis for nanocellulose; green solvent extraction (ethanol, DES); sol–gel extraction of silica/hydrothermal synthesis of HA	Phenolics (olive pomace/olive leaves); nanocellulose (sugarcane bagasse); silica (rice husk) CaCO_3_/hydroxyapatite (eggshells)	Lower solvent use, shorter extraction time; improved yield/purity of phenolics; access to nanocellulose with high crystallinity; high-purity silica or HA with tunable structure	Trade-off between yield and energy consumption; risk of compound degradation; need for removal of solvent/metal residues; biomass variability affects reproducibility
Surface chemistry and bioconjugation	Carbodiimide coupling (EDC/NHS) for amide formation; silanization (e.g., APTES); periodate oxidation; layer-by-layer assembly	Cellulose/nanocellulose; chitosan/polysaccharides; rice husk silica; HA-based composites	Peptide/enzyme/antibody immobilization; tunable surface activity; reusable biocatalysts	Excess grafting reduces biomolecule activity; oxidation or silanization may damage substrates; chemical residues are cytotoxic
Composite formation and additive manufacturing	Polymer blending (PLA, PCL); HA or CaCO_3_ reinforcement (eggshell-derived); ion doping (Zn, Ag); electrospinning (nanofibers); direct ink writing/3D printing of scaffolds	PLA/HA composites (eggshell HA); cellulose-based inks; electrospun nanofibrous membranes	Improved mechanical strength and osteoinductivity; antimicrobial functionality (Ag, Zn); 3D architecture control (porosity, geometry)	Complex processing; cytotoxic ion release if not controlled
Obstacles, limitations, and regulatory aspects	Biomass variability control; standardization (purity, crystallinity, metal content); ISO 10993 biocompatibility; GMP scale-up	—	Reproducibility and clinical safety	Batch variability; scale-up cost and process control; sterilization, long-term in vivo safety
Future directions/research priorities	Open-access materials databases; multifunctional materials; in vivo validation + regulatory roadmaps	Conductive nanocellulose films; HA coatings with antimicrobial and sensing ability	Accelerated translation to clinical products	Translational bottleneck; need for harmonized standards

## Data Availability

No new data were created or analyzed in this study.
